# Shared Mechanisms in Dementia and Depression: The Modulatory Role of Physical Exercise

**DOI:** 10.1111/jnc.70185

**Published:** 2025-08-04

**Authors:** Pedro Borges de Souza, Ana Lúcia S. Rodrigues, Fernanda G. De Felice

**Affiliations:** ^1^ Center of Biological Sciences, Department of Biochemistry Federal University of Santa Catarina Florianópolis SC Brazil; ^2^ Centre for Neuroscience Studies, Department of Biomedical and Molecular Sciences & Department of Psychiatry Queen's University Kingston Ontario Canada; ^3^ D'Or Institute for Research and Education Rio de Janeiro RJ Brazil; ^4^ Institute of Medical Biochemistry Leopoldo de Meis, Federal University of Rio de Janeiro Rio de Janeiro RJ Brazil

**Keywords:** brain atrophy, dementia, depression, HPA axis dysfunction, neuroinflammation, physical exercise

## Abstract

Dementia and depression are two prevalent disorders that warrant significant attention due to their high prevalence and substantial contribution to the global burden of disease. Depression has a high incidence in the elderly and is considered a risk factor for the development of dementia, being a promising target for dementia prevention strategies. Additionally, dementia and depression share common mechanisms through which they manifest pathologically. This review addresses the potential shared mechanisms between dementia and depression, including hypothalamic–pituitary–adrenal (HPA) axis dysfunction, brain atrophy (mainly hippocampus and prefrontal cortex), cognitive decline, and neuroinflammation. It also explores the therapeutic potential of physical exercise in modulating these shared pathways, highlighting its role as a non‐pharmacological intervention for the treatment and prevention of both disorders. The review also explores the muscle–brain crosstalk and the intracellular pathways through which physical exercise exerts its effects.

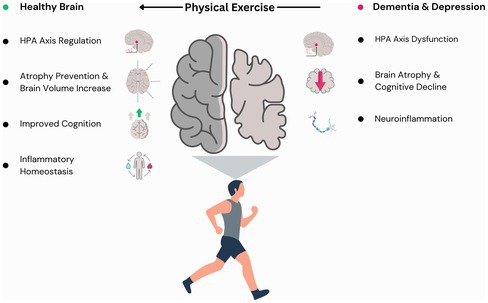

## Graphical abstract

Dementia and depression are two prevalent disorders that warrant attention due to their high prevalence and contribution to the global burden of disease. Depression is a prevalent condition among the elderly population, and it has been identified as a risk factor for dementia, thus becoming an important factor for dementia prevention strategies. Dementia and depression have been shown to share similar mechanisms, including hypothalamic–pituitary–adrenal (HPA) axis dysfunction, brain atrophy, cognitive decline, and neuroinflammation. In this scenario, physical exercise is a promising non‐pharmacological approach for modulating these shared pathways, offering potential benefits in the treatment and prevention of both disorders.

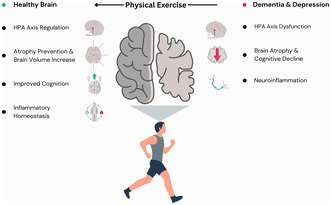

**Abbreviations**
3xTg‐AD: homozygous triple transgenic AD mice
Aβ: amyloid‐β
ABI3: ABI family member 3
ACh: acetylcholine
AChE: acetylcholinesterase
AChEIs: AChE inhibitors
ACTH: adrenocorticotropic hormone
AD: Alzheimer's disease
Akt: protein kinase B
AMPK: AMP‐activated protein kinase
APOE: apolipoprotein E
APP: amyloid precursor protein
APP/PS1: mice harboring expressing a chimeric mouse/human amyloid precursor protein and a mutant human presenilin 1
APPswe/PS1dE9: mice harboring the Swedish APP mutation and deletion of the exon 9 of presenilin‐1
Bax: Bcl‐2–associated X protein
Bcl‐2: B‐cell lymphoma 2
BDNF: brain‐derived neurotrophic factor
CA: cornu ammonis
CNS: central nervous system
CREB: cAMP response element‐binding protein
CRH: corticotropin‐releasing hormone
CRP: C‐reactive protein
CRS: chronic restraint stress
CUS: chronic unpredictable stress
DG: dentate gyrus
ERK: extracellular signal‐regulated kinase
GCs: glucocorticoids
FMRP: fragile X messenger ribonucleoprotein 1
FNDC5: fibronectin type III domain‐containing 5
GSK‐3: glycogen synthase kinase‐3
GWAS: genome‐wide association studies
HIIT: high‐intensity interval training
HPA: hypothalamic–pituitary–adrenal
Iba‐1: ionized calcium‐binding adaptor molecule 1
IFN‐γ: interferon gamma
IL: interleukin
IGF‐1: insulin growth factor 1
MAOIs: monoamine oxidase inhibitors
MCI: mild cognitive impairment
mPFC: medial prefrontal cortex
mTOR: mechanistic target of the rapamycin
NFTs: neurofibrillary tangles
NF‐κB: nuclear factor kappa B
NLRP3: NACHT‐, LRR‐ and pyrin (PYD)‐domain‐containing protein 3
NMDA: N‐methyl‐D‐aspartate
PFC: prefrontal cortex
PGC‐1α: proliferator‐activated receptor gamma coactivator‐1α
p‐GSK‐3β‐Ser9: GSK‐3β phosphorylated at Serine 9
PI3K: phosphatidylinositol 3‐kinases
PKA: protein kinase A
PKC: protein kinase C
PLCG2: phospholipase Cγ2
PSD‐95: postsynaptic density protein 95
PTK2B: protein tyrosine kinase 2β
SNAP91: synaptosome‐associated protein 91
SNRIs: serotonin/norepinephrine reuptake inhibitors
SSRIs: selective serotonin reuptake inhibitors
STAT3: signal transducer and activator of transcription 3
TCA: tricyclic antidepressants
TLR: toll‐like receptor
TNF‐α: tumor necrosis factor alpha
TREM2: triggering receptor expressed on myeloid cells 2
TXNIP: thioredoxin interacting protein
VEGF: vascular endothelial growth factor

## 1 Contextual Background of Dementia and Depression

Dementia is a leading cause of neurological disability worldwide, negatively impacting quality of life, global functioning, physical health, and significantly increasing morbidity and mortality (Nichols et al. 2022; van der Flier et al. 2023). Dementia affected more than 57.4 million people worldwide in 2019, and this number is expected to rise to 152.8 million by 2050 (Nichols et al. 2022). Importantly, the prevalence of dementia is predicted to increase in parallel with rising life expectancy and the aging of the global population (Nichols et al. 2022). Among the causes of dementia, Alzheimer's disease (AD) is the most common, accounting for about half of all cases (van der Flier et al. 2023).

Approximately 35% of dementia cases have been attributed to a combination of risk factors, including education level up to the age of 11–12 years, hypertension in midlife, obesity in midlife, hearing loss, depression in late life, diabetes, physical inactivity, smoking, and social isolation (Livingston et al. 2020). A life course model of dementia shows that some factors can decrease the risk of dementia, as follows: depression or social isolation (4%), physical inactivity (2%), hypertension (2%), diabetes (1%), and obesity (1%) (Dafsari and Jessen 2020; Livingston et al. 2020). Some cases of dementia may therefore be preventable, as some of these factors are modifiable.

The relationship between dementia and depression has been indicated by several studies, since depression is a risk factor for dementia (Byers and Yaffe 2011; Leung et al. 2021). Depression is a prevalent psychiatric disorder worldwide and is among the leading contributors to the global burden of disease (Marx et al. 2023). Approximately 280 million people globally suffer from depression, and it is estimated that 5.7% of adults over the age of 60 experience depression (World Health Organization 2023). The occurrence of depressive symptoms in dementia differs with the severity of the disease, with 38% in mild dementia, 41% in moderate dementia, and 37% in severe dementia (Leung et al. 2021). Depressive symptoms often precede mild cognitive impairment (MCI) and the onset of dementia. Accordingly, depression is considered a prodromal marker for the development of dementia (Elser et al. 2023; Wallensten et al. 2023).

**FIGURE 1:**
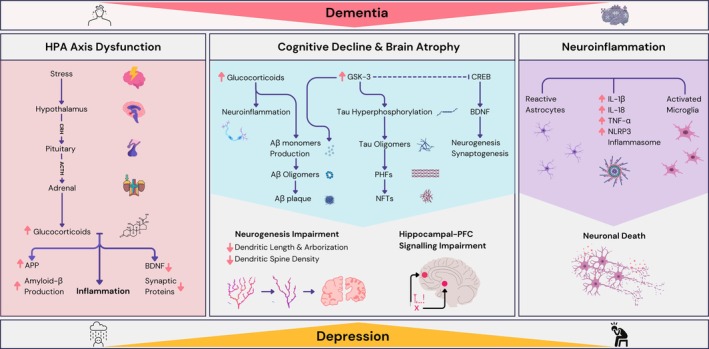
Potential shared mechanisms in dementia and depression. Overactivation of the hypothalamic–pituitary–adrenal (HPA) axis by stressors has been shown to lead to dysfunction and an increase in glucocorticoid levels. This, in turn, has been shown to lead to increases in amyloid precursor protein (APP), amyloid‐β (Aβ) production and inflammation, as well as reduction in synaptic proteins. Neuroinflammation, a hallmark feature of both dementia and depression, involves the activation of microglial and astrocytic cells, leading to the production of pro‐inflammatory cytokines and the activation of the NLRP3 inflammasome, resulting in neuronal death. The interplay between HPA axis dysfunction, neuroinflammation, and elevated protein activity, such as GSK‐3β, contributes to the development of Aβ plaques and neurofibrillary tangles (NFTs). These events ultimately result in impaired neurogenesis, leading to cognitive decline and hippocampal and PFC atrophy, as well as hippocampal‐PFC signaling impairment.

Despite the well‐established connection between depression and dementia, their shared mechanisms have not yet been fully established. However, hypothalamic–pituitary–adrenal (HPA) axis dysfunction, hippocampal atrophy, and neuroinflammation are possible shared mechanisms of these two neurological conditions (Figure 1) (Dafsari and Jessen 2020; Santos et al. 2018; White et al. 2023; Zajkowska et al. 2022).

In this review, we provide evidence on the neuroprotective effect of physical exercise in shared mechanisms of depression and dementia. To this end, a comprehensive search of publications on PubMed using the words physical exercise, dementia, depression, neuroinflammation, hippocampal atrophy, HPA axis, and neuroprotection was performed. Understanding the biological effects and molecular mechanisms of physical exercise can guide public health policies to adopt this non‐pharmacological strategy to prevent and/or mitigate the neural damage induced by dementia and depression.

## 2 Shared Mechanisms in Dementia and Depression

### 2.1 HPA Axis Dysfunction

The HPA axis represents a primary stress response system that enables adaptation to aversive physiological and psychological stimuli. This process is mediated by the release of corticotropin‐releasing hormone (CRH) by neurons in the paraventricular nucleus of the hypothalamus, which stimulates the anterior pituitary gland to produce adrenocorticotropic hormone (ACTH) (dos Santos et al. 2024; Smith and Vale 2006). ACTH, in turn, stimulates the adrenal cortex to synthesize and secrete glucocorticoids (GCs) into circulation. These GCs regulate adaptive physiological changes through ubiquitously distributed intracellular receptors. However, hyperactivation of the HPA axis may contribute to the pathogenesis of several diseases (dos Santos et al. 2024; Smith and Vale 2006).

HPA dysfunction represents a substantial disruption in the hormonal system, occurring in individuals with dementia and depression. In humans, hyperactivation of the HPA axis results in a sustained increase in the GC cortisol (Canet et al. 2019; Ouanes and Popp 2019). High cortisol levels have been correlated with both dementia and depression, as well as the presence of symptoms associated with these conditions, including cognitive and memory deficits (Canet et al. 2019; Ouanes and Popp 2019). In addition, elevated cortisol levels can lead to the onset of dementia and depression (White et al. 2023; Zajkowska et al. 2022). Furthermore, plasma cortisol levels were found to predict hippocampal atrophy, with subjects exhibiting higher cortisol levels demonstrating a more rapid decline in hippocampal volume over time (White et al. 2023). Similarly, an association has been identified between plasma cortisol levels and amyloid‐β (Aβ) brain burden, as measured in vivo (Toledo et al. 2012). The correlation between altered serum cortisol levels of major depressive disorder patients at baseline and serum Aβ42 levels and Aβ40/Aβ42 ratio in the follow‐up represents a risk factor for future onset of AD (Ishijima et al. 2018). Another study also reported elevated salivary cortisol concentrations in the high‐stress, apolipoprotein E (APOE)‐ε4 positive subject group (Peavy et al. 2007).

Disruption of the hippocampal–prefrontal cortex (PFC) circuit is also observed in cases of HPA axis dysfunction and stress (Pizzagalli and Roberts 2022; Yadav et al. 2024). The PFC exerts top‐down control over the hippocampus, thereby enabling cognitive processes, such as spatial working memory and memory consolidation (Jobson et al. 2021; Ruggiero et al. 2021; Yadav et al. 2024). As demonstrated in the literature, the deactivation of the PFC during the learning process results in impaired task performance in both spatial and non‐spatial learning domains (Yadav et al. 2024). The diminished activation of the ventrolateral PFC and dorsolateral PFC has been shown to intensify the symptoms of depression (Disner et al. 2011). Additionally, medial PFC (mPFC) circuits in AD uniquely disconnect from the hippocampus, potentially impacting symptomatic memory deterioration (Jobson et al. 2021). Two studies using the chronic unpredictable stress (CUS) depression model observed the effects of stress on the hippocampal‐PFC circuit. One study found a weaker long‐term potentiation induction and reduced theta phase synchrony in the ventral cornu ammonis (CA) 1‐to‐mPFC circuit compared with the control group (Zheng and Zhang 2015). Similarly, another study found that local field potential oscillation was lower in CUS rats than in the control group in the beta and gamma frequency ranges. The research also found that either high‐frequency stimulation with low‐intensity or low‐frequency stimulation with high intensity in the ventromedial PFC induced an antidepressant‐like effect in rats subjected to CUS (Jia et al. 2019). Furthermore, the administration of GC receptor agonist into the mPFC of rats significantly impaired working memory performance, as assessed by reduced task accuracy (Barsegyan et al. 2010). A study that subjected mice to maternal separation during the postnatal days 2–20 reported a reduction in the expression of pre‐ and postsynaptic protein in inhibitory neurons, as well as a decrease in the number of GAD67‐positive interneurons and inhibitory synapses in the mPFC of 9‐week‐old mice. In addition, maternal separation has been demonstrated to impair social behavior related to social recognition, a process closely linked to the mPFC (Ohta et al. 2020).

Other in vitro and in vivo studies have also reported the effects of GCs and stress in models of dementia and depression. In dissociated cell cultures of rat hippocampus, elevated levels of GCs have been shown to accelerate hippocampal atrophy and enhance Aβ toxicity (Goodman et al. 1996). Exposure to GC has also been demonstrated to increase Aβ production by upregulating the expression of amyloid precursor protein (APP) and β‐site APP‐cleaving enzyme 1 in primary cultures of astrocytes and astrocytes of both normal and middle‐aged mice (Wang et al. 2011). Elevation of corticosterone levels induced by maternal separation in rats has also been associated with increases in Aβ40 and Aβ42 levels and decreases in brain‐derived neurotrophic factor (BDNF) and synaptic proteins synaptophysin and postsynaptic density protein 95 (PSD‐95) immunocontent, in addition to a decrease in cell number in the hippocampus (Martisova et al. 2013). Further corroborating the relationship between disrupted HPA axis and dementia, homozygous triple transgenic AD (3xTg‐AD) mice, which contain three mutations associated with familial AD (PS1M146V, APPswe, and tauP301L) have been shown to exhibit an activated HPA axis, with altered mRNA levels of mineralocorticoid and GC receptors in the hippocampus (Hebda‐Bauer et al. 2012). Chronic stress and GC treatment also induce alterations in the content and phosphorylation of the tau protein in the hippocampus and PFC, which is the first step in the formation of neurofibrillary tangles (NFTs) (Carroll et al. 2011; Sotiropoulos et al. 2011; Yang et al. 2014). It is important to highlight that the Tg2576 mice, which exacerbate Aβ accumulation, and the P301S mutation (PS19) mice model, which displays tau hyperphosphorylation, exhibit an elevated HPA stress response in the absence of stress (Carroll et al. 2011). In summary, evidence from human, in vitro, and in vivo studies indicates that HPA axis hyperactivity is a probable predictor of AD development. Moreover, hyperactivation of the HPA axis during the course of the disease contributes to the subsequent deterioration of cognitive function, the advancement of the disease, and the intensification of clinical symptoms over time.

### 2.2 Cognitive Decline and Brain Atrophy

Cognitive decline is an intrinsic feature of both dementia and depression, serving as a defining characteristic of these disorders (Hugo and Ganguli 2014; Yin et al. 2024). The presence of dementia or MCI is a significant risk factor for the development of depressive symptoms. Conversely, a considerable proportion of individuals with depression exhibit signs of MCI, with depressive symptoms being particularly associated with amnestic MCI (Ismail et al. 2017). In addition, individuals with β‐amyloid‐positive cerebrospinal fluid and a history of depressive symptoms are more susceptible to cognitive decline and the development of AD, as evidenced by faster brain atrophy and cognitive decline (Zhang, Tan, et al. 2025).

In this scenario, hippocampal atrophy may account for the observed association between cognitive impairment in dementia and depression. Hippocampal atrophy is one of the primary and earliest markers observed in dementia, particularly in AD (van de Pol et al. 2006). Similarly, reduced hippocampal volume is probably the most commonly observed structural neuroimaging finding associated with depression (Santos et al. 2018). Accordingly, the hippocampus is a brain structure directly linked to cognition and mood. Particularly, the dentate gyrus, which is part of the hippocampal formation, possesses the capacity for continuous neurogenesis, whereby new neurons develop into mature neurons and functionally integrate into existing neural circuits (Anacker and Hen 2017).

The potential mechanisms underlying hippocampal atrophy can be related to stress‐induced increases in GCs, via activation of the HPA axis, and reductions in neurotrophic factors, which together lead to reduced hippocampal neurogenesis. This, in turn, leads to impairments in hippocampal synaptic plasticity and morphological neuroplasticity, ultimately culminating in cognitive deficits (Wiels et al. 2020). For example, a study conducted on subjects with amnestic MCI demonstrated that plasma cortisol levels were predictive of greater hippocampal atrophy. Participants with higher cortisol exhibited a faster decline in hippocampal volume over time (White et al. 2023). Protocols involving chronic stress or corticosterone/GC treatment in rodents have demonstrated a decrease in neurogenesis and gliogenesis, deficits in cell proliferation and differentiation, and cell survival in the hippocampus (Duman and Aghajanian 2012; Leschik et al. 2021). In addition, stress exposure can lead to a reduction in apical dendritic length and branching, as well as a decline in spine density in mPFC, CA1, and CA3 pyramidal neurons in the hippocampus (Duman and Duman 2015; Qiao et al. 2016). Imaging studies have also demonstrated an association between a history of early‐life stress and volumetric decline in the hippocampus and mPFC (Belleau et al. 2019).

Another important mechanism contributing to neurogenesis impairment, which in turn results in cognitive decline and hippocampal atrophy in dementia and depression, is the overexpression/lower inhibition of glycogen synthase kinase‐3 (GSK‐3). GSK‐3 is a ubiquitous protein kinase with a multitude of functions, particularly in the neurons, and is presented in two isoforms: GSK‐3α and GSK‐3β. GSK‐3β can be phosphorylated at Serine 9 (p‐GSK‐3β‐Ser9), which inhibits the ability of GSK‐3 to phosphorylate its primed substrates and exert its effects (Li and Jope 2010). Indeed, the overexpression of GSK‐3 has been linked to several pathological processes, including tau hyperphosphorylation, regulation of γ‐secretase, and increases in Aβ production (Lauretti et al. 2020). It has also been observed to induce inflammatory responses and memory impairment (Li and Jope 2010; Zhao et al. 2024).

A study conducted on elderly subjects observed an increase in GSK‐3β activity in patients with AD presenting depressive symptoms or in those exhibiting mild dementia. These findings are accompanied by a reduction in BDNF levels in platelet‐rich plasma of patients with AD at moderate‐to‐severe stages of dementia or in AD without depressive symptoms. Moreover, elevated GSK‐3β activity in the peripheral blood of patients with AD has been demonstrated to be positively correlated with the severity of dementia. A lower level of phosphorylated GSK‐3β was observed in patients with depressive disorders, and this was found to be associated with a higher severity of depression (Pláteník et al. 2014). In contrast, another study conducted with elderly patients with depression revealed significantly lower p‐GSK‐3β levels and lower GSK‐3β ratio, suggesting increased GSK3B activity. Moreover, heightened GSK‐3β activity has been documented in patients experiencing severe depressive episodes and cognitive impairment (Diniz et al. 2011). In line with these observations, GSK‐3β mRNA expression in the postmortem hippocampus was found to be significantly elevated in the depression group when compared with the control group. These findings also revealed a substantial correlation between GSK‐3β mRNA expression levels and nitric oxide synthase 1 (Oh et al. 2010). Additionally, rodent models have linked the overexpression or lower inhibition of GSK‐3 with dementia models, particularly AD models, and depressive‐like behavior (Cheng et al. 2016; Rodríguez‐Matellán et al. 2020).

### 2.3 Neuroinflammation

Neuroinflammation in the central nervous system (CNS) is a complex phenomenon that encompasses a multitude of physiological responses involving microglial and astrocytic cells, as well as the release of pro‐inflammatory factors. Microglial activation represents an initial defense mechanism that occurs in response to an insult to the CNS. However, in conditions of chronic inflammation and sustained microglial activation, the activation of inflammatory signaling pathways results in decreased levels of BDNF, thereby impairing neuroplasticity‐related mechanisms (Ahmad et al. 2022; Kouba et al. 2024). Therefore, neuroinflammation, particularly in brain areas involved in learning and memory, has been implicated in cognitive impairment that occurs in patients with dementia and/or depression. The activation of the NACHT‐, LRR‐, and pyrin (PYD)‐domain‐containing protein 3 (NLRP3) inflammasome in microglia represents a key event in neuroinflammation due to increased levels of interleukin (IL)‐1β and IL‐18 that perpetuate the inflammatory process in the CNS (Kouba et al. 2022).

In individuals with MDD, the increase in pro‐inflammatory mediators, mainly C‐reactive protein (CRP), tumor necrosis factor alpha (TNF‐α), IL‐1β and IL‐6, are often elevated in the peripheral blood (Miller and Raison 2016). Similarly, increased levels of IL‐1β and IL‐6 have been reported in the cerebrospinal fluid of AD individuals (Blum‐Degena et al. 1995). Furthermore, the process of aging is accompanied by an increase in the permeability of the blood–brain barrier. This increase in permeability facilitates the infiltration of peripheral immune cells into the CNS (Knox et al. 2022).

Increased levels of pro‐inflammatory cytokines in the hippocampus and PFC have been reported, both in animal and human studies (Belleau et al. 2019; Mittli 2023). Imaging studies in humans experiencing a major depressive episode observed enhanced neuroinflammation in the hippocampus and mPFC compared with healthy controls (Holmes et al. 2018; Setiawan et al. 2015). Similarly, enhanced neuroinflammation in the hippocampus, frontal cortex, and temporal cortex, as well as impaired memory in the attention and delayed domains, was observed in never‐medicated patients with depression compared with healthy controls (Li et al. 2018). To assess the significance of the anti‐inflammatory cytokine IL‐10 and the inflammatory imbalance, Yang et al. (2021), employed an IL‐10 *knockout* mouse model. The results showed that the *knockout* mice exhibited depression‐ and anxiety‐like behavior, as compared with their littermates. In addition, these animals showed a decrease in the expression of transient receptor potential canonical 5, a cationic membrane channel that induces a Ca^2+^‐permeability in mPFC, as well as an increase in IL‐1β and IL‐6 levels (Yang et al. 2021). In mice that exhibited a LPS‐induced depressive‐like behavior, the natural non‐steroidal isoflavonoid formononetin reversed the depressive‐like behavior, rebalanced microglia polarization, inhibited NLRP3 inflammasome, and enhanced microglial autophagy level in the PFC (Peng et al. 2025). LPS administration also increased IL‐1β and IL‐6 levels and reduced *Bdnf* mRNA in the hippocampus and PFC of C57BL/6 mice (Rezaei et al. 2024). Interestingly, chemogenetic activation of the hippocampus‐mPFC pathway improved LPS‐induced cognitive dysfunction in C57BL/6 mice (Ge et al. 2023).

It has been well established that peripheral pro‐inflammatory mediators may reach the CNS, leading to microglial activation (Colonna and Butovsky 2017; Zrzavy et al. 2019). The degree and nature of microglial activation depend on the nature and severity of the insult. In general, microglia can assume a pro‐inflammatory phenotype characterized by the thickening and retraction of its branches following exposure to pathogenic stimulus. Under this condition, microglia release pro‐inflammatory cytokines and reactive oxygen species, leading to a cascade of inflammatory events that include astrocytic activation and neuronal injury (Kouba et al. 2022). These events are reported to occur in dementia and depressive states. However, in AD, the role of microglial activation in the pathogenesis of the disease has been debated. Baligács et al. (2024) provided some insight into this topic by showing that depletion of microglia before Aβ plaque deposition results in a decrease in plaque numbers and neuritic dystrophy, indicating their role in plaque initiation. On the contrary, in later stages, activated microglia compact plaques, limiting their toxicity, a finding that suggests a protective role for microglia in later stages of the disease (Baligács et al. 2024). A recent study conducted by Liu, Wang, et al. (2025), Liu, Xiao, et al. (2025) and Liu, Zhao, et al. (2025) showed that depression exacerbates AD pathology. In a 5xFAD model, mice that experienced learned helplessness‐induced depressive‐like behavior at a young age exhibit a significantly more pronounced Aβ burden, defined as Aβ deposition in the cortex and dentate gyrus, as well as Aβ‐containing exosome spreading and cognitive deficit. The energy metabolism in microglia of depressed AD mice is reprogrammed, resulting in the production of a significant amount of lactate. This, in turn, caused the upregulation of outward‐rectifying potassium channel Kv1.3 activity, facilitating the release of Aβ‐containing exosome from microglia in the vicinity of Aβ plaque, thereby propagating AD pathology (Liu, Wang, et al. 2025).

Indeed, research has demonstrated a correlation between microglial activation and the formation of Aβ plaques. This association is believed to occur through a series of complex mechanisms, including the increased secretion of Aβ fragments, the release of modulatory proteins, such as γ‐secretase, and the release of agents that facilitate the initial aggregation of Aβ, such as iron. The activation of microglia has been demonstrated to promote tau hyperphosphorylation, which in turn induces the formation of tau aggregates and leads to the formation of NFTs (Heneka et al. 2024; Zhang, Xiao, et al. 2023). There is also evidence that numerous inflammation‐risk genes have been identified as exacerbating neuroinflammation in dementia. Genome‐wide association studies (GWAS) have identified multiple genes that have been demonstrated to promote microglial activation and increase microglial response, including APOE4, phospholipase Cγ2 (PLCG2), triggering receptor expressed on myeloid cells 2 (TREM2), protein tyrosine kinase 2β (PTK2B), CD33, and ABI family member 3 (ABI3). These genes have been associated with the development of dementia, suggesting that microglia‐mediated innate immune response contributes directly to the development of this condition (Heneka et al. 2024; Newcombe et al. 2018; Sims et al. 2017).

The activation of microglia has been demonstrated to induce NF‐kB signaling and result in NLRP3 inflammasome activation. NLRP3 inflammasome is a multi‐molecule complex containing cytosolic NLRP3, adaptor protein ASC, and pro‐caspase‐1 precursor. When NLRP3 is activated, it modulates the activation of caspase‐1, which, in turn, promotes the maturation of pro‐IL‐1β and pro‐IL‐18 into the active form (IL‐1β and IL‐18) in microglia. The excessive secretion of pro‐inflammatory cytokines, a consequence of this process, contributes to the onset of dementia and depression (H. Wang et al. 2022). Furthermore, activated microglia have been shown to secrete extracellular vesicles that have been identified as responsible for the transportation and distribution of soluble toxic Aβ peptides. These Aβ peptides have been found to prompt the activation of toll‐like receptor (TLR) 4, which, in turn, initiates the activation of the NLRP3 inflammasome. This, in turn, results in the release of inflammatory cytokines and the subsequent spread of neuroinflammation, leading to the development of dementia (Trotta et al. 2018).

In the same way, certain complement components, including C1q, C3, C4, and C5b‐C9, have been identified as potential contributors to dementia and depression (Pillai 2022; Wu et al. 2019; Yang et al. 2020). The excessive activation of these complement components has been observed to result in the accumulation of Aβ plaques and NFT, which can lead to neuroinflammation and neurotoxicity (Yang et al. 2020). Complement receptor activation has been observed to synergize with other innate immune signaling pathways, such as TLRs, which have been implicated in the pathophysiology of dementia and depression (Alawieh et al. 2021; Heneka et al. 2024). Observations have indicated that the suppression or reduction of the expression of these complement components offers a neuroprotective effect, manifested as a prevention of neurodegeneration, loss of dendritic spines, and reduction of excessive microglial cell‐mediated synapse loss. This suppression is accompanied by a reduction in the activation of microglia and astrocytes (Gao et al. 2023; Heneka et al. 2024; Yang et al. 2020; Zhang, Xiao, et al. 2023). Consequently, the modulation of complement cascades emerges as a significant therapeutic strategy in the management of dementia and depression.

## 3 Possible Therapeutic Approaches for the Treatment of Dementia and Depression

### 3.1 Pharmacological Strategies for Management

In light of the current global prevalence of dementia and depression, coupled with projections indicating an increased incidence of these disorders in the coming decades, a more profound comprehension of the correlation between these two conditions is imperative for the advancement of innovative strategies to prevent and treat these disorders. It is crucial to acknowledge that dementia is not a curable condition. Additionally, approximately 30% of patients do not achieve remission from depression, even after multiple treatment attempts. Some studies have already attempted to understand current pharmacotherapy, as well as new drug candidates in the management of dementia (reviewed in Zhang, Zhang, et al. 2024) and depression (reviewed in Cui et al. 2024).

The current pharmacotherapy for dementia is predicated on the utilization of acetylcholinesterase (AChE) inhibitors (AChEIs), which serve to enhance postsynaptic stimulation through the modulation of acetylcholine (ACh) and N‐methyl‐D‐aspartate (NMDA) receptor antagonists, which modulate glutamate transmission and dopamine receptors (Zhang, Zhang, et al. 2024). Conversely, the current pharmacotherapy for depression is predicated on the monoamine theory, which aims to augment monoamines (e.g., serotonin, dopamine, and norepinephrine) in the synaptic cleft. The most prevalent classes of antidepressants include tricyclic antidepressants (TCA), selective serotonin reuptake inhibitors (SSRIs), serotonin/norepinephrine reuptake inhibitors (SNRIs), and monoamine oxidase inhibitors (MAOIs) (Cui et al. 2024). Notably, studies have indicated that antidepressant treatment in subjects with dementia may enhance their well‐being and/or decelerate the progression of the disorder (Bartels et al. 2018; Khoury and Grossberg 2019; Zhang, Zheng, and Zhao 2023). However, these associations have not been consistently replicated in other studies (Bouter and Bouter 2022; Qin et al. 2022).

Research has demonstrated that SSRIs enhance serotonin signaling and have been linked to reduced Aβ plaque levels and burden in elderly individuals and murine models of AD (Cirrito et al. 2011; Sheline et al. 2014). SSRIs have also demonstrated the capacity to decrease plasma tau levels and restore metabolic activity in the dorsal raphe nucleus in patients with AD (Terstege et al. 2025). Moreover, subjects who received antidepressant treatment for 5 years exhibited diminished amyloid load (Cirrito et al. 2011). The soluble and insoluble forms of Aβ and tau are pivotal in determining the cognitive outcomes of AD. Antibodies designed to enhance the clearance of Aβ from the brain have been evaluated; however, it is important to note the risks associated with amyloid‐related imaging abnormalities and the associated treatment costs (Boxer and Sperling 2023). The inhibition of GSK‐3 also has a potential target due to its role in increasing Aβ load and tau hyperphosphorylation. Several studies using transgenic mice models of AD showed a cumulative reduction of tau hyperphosphorylation and alleviation of the AD cognitive/behavioral phenotype through GSK‐3 inhibition (Basheer et al. 2023). The silencing of GSK‐3β in the dentate gyrus has been observed to induce an antidepressant‐like effect in mice (Omata et al. 2011), and the SSRI escitalopram may protect from tau hyperphosphorylation by activating protein kinase A (PKA) and involving the protein kinase B (Akt)/GSK‐3β signaling pathway (Wang et al. 2016). In this regard, the inhibition of GSK‐3 results in a reduction of both tau hyperphosphorylation and Aβ production which, in turn, leads to a decrease in neuroinflammation (Basheer et al. 2023).

Another potential mechanism by which antidepressants may exert their effects in the context of cognitive decline involves the enhancement of cognitive reserve. The concept of cognitive reserve was initially developed in the context of aging and dementia, but it has also been used recently as a potential protector against depression (Camprodon‐Boadas et al. 2024; Stern 2012). Cognitive reserve is defined as the capacity of the brain to withstand a greater degree of adversity, whether from aging, diseases, or mental disorders, before the onset of clinical symptoms (Stern 2009). It has been observed that depressed subjects who have higher levels of cognitive reserve demonstrate enhanced cognitive performance when compared to the control group (Lin et al. 2020; Venezia et al. 2018). The hypothesis that cognitive reserve levels can be induced through the use of antidepressants is one that requires further investigation. Antidepressants have been demonstrated to induce neuroplasticity and cognition, particularly through chronic administration (Boldrini et al. 2009; Rosas‐Sánchez et al. 2024). Neuroplasticity has been demonstrated to be directly associated with increased cognitive reserve, as evidenced by enhanced brain network dynamics (Savarimuthu and Ponniah 2024). In addition, research findings indicate that antidepressant administration enhances brain network dynamics in patients diagnosed with depression, in comparison with individuals who do not use antidepressants and those in the control group (Broeders et al. 2024). The enhanced resilience of individuals with higher levels of cognitive reserve, as evidenced by their ability to withstand more adversity before exhibiting clinical symptoms, can be attributed to a more efficient and flexible utilization of brain networks (Stern 2009, 2012). However, contradictory evidence suggests that cumulative benzodiazepine and antidepressant use in older adults without baseline cognitive impairment may lead to cognitive decline (Chandramouleeshwaran et al. 2024). Antidepressant use has been associated with accelerated cognitive decline in subjects with dementia compared with non‐use. Furthermore, higher doses of SSRIs have been associated with an elevated risk of severe dementia (Mo et al. 2025).

Neuroinflammation represents a prominent target in the context of both depression and dementia. This hypothesis that antidepressant agents can modulate inflammatory responses has been postulated. For instance, SSRIs and SNRIs have been shown to reduce the expression of pro‐inflammatory cytokines, such as IL‐6 and TNF‐α, in depressed subjects (Patel et al. 2023). In addition, in vitro and in vivo studies have demonstrated the capacity of SSRIs to reduce pro‐inflammatory cytokines, such as IL‐6, TNF‐α, interferon gamma (IFN‐γ), and IL‐1β, while often increasing levels of the anti‐inflammatory cytokine IL‐10 (Dafsari and Jessen 2020; Patel et al. 2023). Furthermore, the SSRI fluoxetine has been observed to inhibit the NLRP3 inflammasome via the Akt/signal transducer and activator of transcription 3 (STAT3) and extracellular signal‐regulated kinase (ERK)/STAT3 pathways in astrocytes derived from a sleep‐deprived mouse model (Xia et al. 2018). It is noteworthy that several drugs studied for the treatment of dementia have, at least in part, anti‐inflammatory activity (Zhang, Zhang, et al. 2024).

Despite the availability of treatments and attempts at new therapies, it is important to acknowledge that there is no cure or effective treatment for dementia (Zetterberg and Bendlin 2021). Similarly, current depression therapies have several limitations. These include the late onset of antidepressant effect, the adverse effects associated with the treatment, and the failure of approximately 30% of patients to remit from depression, even after multiple treatment attempts (Otte et al. 2016; Marx et al. 2023). In this scenario, new approaches have also been investigated for the treatment and/or prevention of both dementia and depression. Among these approaches, physical exercise has emerged as a prominent strategy.

### 3.2 Physical Exercise

Physical exercise represents a powerful non‐pharmacological strategy that is readily accessible to the general population. It is well documented that this strategy enhances overall quality of life and mitigates the impact of several diseases (Pedersen and Saltin 2015). In particular, physical exercise has been demonstrated to have a beneficial impact on mental health, with the potential to improve outcomes in a range of neurological and psychiatric disorders, including dementia and depression (de Almeida et al. 2020; Pearce et al. 2022; de Souza et al. 2023). Physical exercise has also been identified as a potential therapeutic tool for the prevention, delay, and treatment of cognitive decline, which is a hallmark of both depression and dementia. This is due to the fact that physical exercise has been shown to improve overall cognitive function (Aghjayan et al. 2022; Erickson et al. 2019; Garrett et al. 2024). However, there is no unanimous consensus among researchers regarding the impact of physical exercise on cognitive function. While some studies have observed a beneficial effect, others have either failed to detect an influence or have documented only a minor effect (Ciria et al. 2023; Liu, Zhao, et al. 2025). The discrepancies in findings regarding the effects of physical exercise may be due to the fact that different exercise training regimens elicit variable responses, while genetic and environmental factors can also be identified as contributing to the variability in the response to exercise training (Ross et al. 2019). In contrast, physical inactivity has been linked to an increased risk of developing depression and dementia (Y. Huang et al. 2020; Livingston et al. 2020).

Research has demonstrated that physical exercise effectively mitigates a range of physiological comorbidities associated with dementia and depression, including cardiovascular disease, obesity, diabetes, and hypertension (Pedersen and Saltin 2015; Zouhal et al. 2022). These health outcomes have the potential to occur prior to the onset of psychiatric diseases, thereby contributing to an increase in allostatic load. This, in turn, has been demonstrated to contribute to the development of dementia and depression. Accordingly, the practice of physical exercise has been shown to exert a preventive effect on the development of dementia and depression (De Felice et al. 2022; Livingston et al. 2020).

During physical exercise, the synthesis and release of exerkines, such as myokines (including irisin, cathepsin B, and IL‐6), neurotrophic factors (such as BDNF, insulin growth factor 1 [IGF‐1] and vascular endothelial growth factor [VEGF]), and adipose tissue‐derived molecules (adiponectin) may contribute to the onset of neuromodulatory cascades (Pedersen 2019). These myokines and neurotrophic factors can act directly on the CNS, contributing to synaptic plasticity, neuronal differentiation, and neuronal health. Additionally, they exert indirect beneficial effects on the CNS by modulating the microbiota‐gut‐brain axis (de Souza et al. 2023).

#### 3.2.1 Physical Exercise and HPA Axis Dysfunction in Dementia and Depression

Several studies have sought to elucidate the mechanisms through which physical exercise exerts its beneficial effects on health. It is well reported that physical exercise enhances the levels of circulating GCs, in both humans and rodents, independently of gender (Caplin et al. 2021; Chen et al. 2017).

It is reported that a variety of acute exercise paradigms in humans (treadmill running, cycling, rowing, and competitive marathon running) and acute treadmill running exercise in rodents significantly increase cortisol and corticosterone levels, respectively (Chen et al. 2017). It is important to note that the greater the intensity of physical exercise, the greater the increase in circulating GCs. In a randomized clinical trial, young, healthy male participants were subjected to exercise on a treadmill at either 30%, 50%, or 70% of their heart rate reserve for 30 min. Subsequently, 45 min later, they were subjected to a psychosocial stress induction task (Trier Social Stress Test). It has been demonstrated that higher levels of salivary cortisol are associated with higher‐intensity exercise. However, vigorous exercise (70% of heart rate reserve) elicited a dampened salivary cortisol response to the psychosocial stressor task, marked by lower total cortisol levels, diminished reactivity, and faster recovery to baseline values as compared to less intense exercise (Caplin et al. 2021). These effects may be attributed to a GC negative feedback mechanism, which results in a dampened cortisol response when the subjects are subject to the stressor. Other compelling evidence of the influence of physical exercise on cortisol levels is presented by a systematic review that analyzed 10 randomized controlled trials examining the effect of physical exercise on cortisol levels and sleep quality. The findings indicated that physical exercise demonstrated moderate evidence of reducing cortisol levels, as well as low‐certainty evidence of improving sleep quality. However, these results need attention, as the majority of studies evaluated physical exercise in breast cancer and included a limited number of males and no older adults (De Nys et al. 2022). Nevertheless, another systematic review from the same research group assessed the impact of physical activity on elderly subjects and demonstrated low‐ to moderate‐quality evidence that regular physical activity beneficially reduces cortisol levels and increases dehydroepiandrosterone, a GC that counterbalances many of the negative effects of cortisol (De Nys et al. 2022). A systematic review also revealed that physical exercise reduces cortisol levels in subjects with depression. Cortisol levels were observed to decrease in the exercise group, and aerobic exercise was identified as the most effective type of physical exercise for reducing cortisol levels, with the greatest benefits observed when performed five times per week (Beserra et al. 2018).

The mechanisms by which physical exercise regulates the HPA axis and the release of GC have been extensively studied in several rodent models of stress. In a chronic restraint stress (CRS) model, 14 days of treadmill exercise prevented anxiety‐like behavior through the maintenance of normal neural activity and axonal myelination in the mPFC. These results are accompanied by a modulation of the fragile X messenger ribonucleoprotein 1 (FMRP)/mechanistic target of the rapamycin (mTOR) pathway in the mPFC. Specifically, treadmill running exercise activates mTOR and mediates the brain RNA methylation, which inhibits the expression of FMRP (Yan et al. 2023). Similarly, another study from the same research group demonstrates that treadmill exercise also prevents anxiety‐like behavior induced by CRS through an increase in circulating lactate and potentiating the lactylation of multiple synaptic proteins, particularly synaptosome‐associated protein 91 (SNAP91) in the mPFC, which is a critical molecule for synaptic functions (Yan et al. 2024). Furthermore, swimming exercise has been demonstrated to alleviate chronic unpredictable mild stress‐induced depressive‐like behavior, enhance neuroplasticity‐related proteins and microtubule‐related proteins, and reduce neuroinflammation in the hippocampus (Xie et al. 2022).

It also has been shown that physical exercise regulates CUS‐impaired oligodendrocytes and CUS‐induced microglial activation. Treadmill running exercise alleviates anhedonic‐like behavior and decreases in mPFC volume, as well as promoting differentiation of oligodendrocytes and myelin‐forming ability in the mPFC in a CUS rat model of depression (Luo et al. 2019). Treadmill running exercise also prevents CUS‐induced anhedonic‐ and depressive‐like behavior while reducing microglial activation in the murine hippocampus (L. Liu et al. 2024; Xiao et al. 2021). These effects of treadmill exercise may be triggered by adiponectin/AdipoR1‐mediated activation of the AMP‐activated protein kinase (AMPK)‐nuclear factor kappa B (NF‐κB)/STAT3 signaling pathway in the hippocampus (Liu et al. 2024). In 8‐week‐old C57BL6/J mice, voluntary running wheel in a stress‐free environment enhanced resilience to CRS, as evidenced by reduced activation of the HPA axis and corticosterone response. These results were accompanied by an interaction of the mPFC–ventral hippocampus pathway, in which activation predominantly of hippocampal GABAergic neurons and suppression of the activity in the mPFC were shown. In addition, chemogenetic inhibition of the ventral hippocampus dissipated the antidepressant and anxiolytic effects of voluntary running wheel pretreatment (Hwang and Cho 2025).

#### 3.2.2 Impact of Physical Exercise on Brain Atrophy and Cognitive Decline in Dementia and Depression

As previously described, physical exercise has been recognized as a strategy to afford beneficial effects on global cognition in individuals with dementia and depression (Aghjayan et al. 2022; Garrett et al. 2024). This effect can be attributed to different mechanisms through which physical activity influences the CNS. One of these mechanisms that has been extensively investigated is how physical exercise increases hippocampal volume and prevents its decline in the course of dementia and depression.

##### 3.2.2.1 Evidence of the Effect of Physical Exercise on Cognition and Hippocampal Volume in Human Studies

Some meta‐analyses have identified a correlation between physical exercise and an increase or prevention of decline in total or regional hippocampal volume in humans. These meta‐analyses have considered a diverse range of samples, including adults and the elderly, individuals with and without cognitive decline, those with and without dementia or depression, and different approaches to physical exercise (Huang et al. 2022; Kress et al. 2024; Wilckens et al. 2021).

Several human studies using different physical exercise regimens have demonstrated these beneficial effects. Research has shown that aerobic fitness training increases both gray and white matter regions in healthy sedentary older subjects (Colcombe et al. 2006). Additionally, high‐intensity interval training (HIIT) has been shown to enhance hippocampal functionality and mitigate age‐related volumetric decline in certain brain regions, including the hippocampus. HIIT has also been shown to enhance functional connectivity between different neural networks in elderly subjects without cognitive decline (Blackmore et al. 2024). Similarly, aerobic exercise has been demonstrated to increase total left hippocampal and left CA4‐dentate gyrus (DG) volumes and to prevent age‐related volume decline in sedentary healthy older adults when compared to control subjects who did not engage in exercise (Frodl et al. 2020). Another study found that physical activity was associated with an increase in hippocampal, parahippocampal, entorhinal, and fusiform gyrus volumes in cognitively intact older adults (Aslanyan et al. 2023). Moreover, an increase in the hippocampal volumes of healthy older women who perform strength training exercises has been demonstrated (Kim et al. 2017). In addition, greater physical activity in older adults was found to be a significant predictor of higher frontal, occipital, entorhinal, and hippocampal volumes 9 years later (Erickson et al. 2010). In healthy older adults who performed aerobic exercise, increases in anterior hippocampal volume and improved spatial memory were associated with increases in BDNF levels. This increase in hippocampal volume effectively reverses the age‐related loss of volume by approximately 1–2 years (Erickson et al. 2011).

A similar increase in the volume of the dorsolateral PFC and posterior cingulate/precuneus cortex has been observed in healthy elderly individuals following physical exercise (Ji et al. 2017). Moderate‐ and high‐intensity physical exercise has been shown to increase PFC activity during cognitive load induced by dual task in healthy young adults (Kimura et al. 2022). A 10‐min exercise session on a cycle ergometer at a low intensity resulted in enhanced cognitive performance and increased arousal levels. This increase in arousal levels was positively correlated with enhanced cognitive performance in the left dorsolateral PFC and the frontopolar area (Byun et al. 2014).

Research has demonstrated that aerobic exercise enhances left, right, and total hippocampal volumes in elderly women with probable MCI when compared with the balance and tone training group (ten Brinke et al. 2015). A study revealed that the substantial volume of self‐reported exercise at baseline has been linked to a decelerated decline in multiple brain regions, including hippocampal volume, and a reduced accumulation of brain Aβ at preclinical disease stages in individuals with autosomal dominant AD in the follow‐up (Sewell et al. 2024). The combination of physical exercise with visually stimulating computer games showed a significant increase in the volume of specific brain regions, namely the left hippocampal subfields CA1, CA4/DG, and subiculum, as well as a significant rise in serum BDNF levels in individuals with Parkinson's disease who underwent a 6‐week training program (Schaeffer et al. 2022).

Indeed, these effects of increasing or preventing hippocampal volume loss can be attributed to the production of BDNF, a mediator of neurogenesis in the hippocampus. A meta‐analysis of controlled trial studies demonstrated a significantly higher BDNF response to acute aerobic exercise and to aerobic or strength training programs in healthy elderly individuals and elderly individuals with different pathologies. It is noteworthy that moderate‐intensity exercises have been shown to be more effective in promoting an increase in peripheral BDNF levels in the elderly (de Coelho et al. 2013).

##### 3.2.2.2 Evidence of the Effect of Physical Exercise on Cognition in Animal Studies

Several studies in rodents have investigated the effects of physical exercise on hippocampal volume and neurogenesis. A study utilizing 5xFAD mice, a transgenic model of AD, demonstrated that 3 h per day in cages equipped with running wheels induces adult hippocampal neurogenesis and enhances cognition while reducing Aβ load and increasing levels of BDNF, IL‐6, fibronectin type III domain‐containing 5 (FNDC5), and synaptic markers (Choi et al. 2018). In the early stage of AD in the 3xTg‐AD mouse model, 12 weeks of treadmill running exercise reversed the cognitive impairment and significantly improved the tau pathology, along with the suppression of the decreased PSD‐95 and synaptophysin in the hippocampus and cortex. Moreover, in the advanced AD stage of the 3xTg‐AD mice model, 12 weeks of treadmill running exercise reversed the cognitive impairment and significantly improved the Aβ and tau pathology, along with the suppression of the decreased synaptic proteins and BDNF in the hippocampus and PFC (Cho et al. 2015). Eight weeks of aerobic exercise exerted a significant impact on the enhancement of spatial cognition and learning and memory abilities in both wild‐type and APP/PS1 mice when compared to their respective sedentary controls. Aerobic exercise has also been shown to induce an upregulation of the Xc^−^/glutathione peroxidase 4 pathway, which can reverse the lipid peroxidation process, thereby inhibiting ferroptosis in the PFC of the APP/PS1 mice (Li et al. 2024). Voluntary running wheel has been demonstrated to rescue memory impairment in APP/PS1 mice by reducing Aβ plaques, enhancing myelination, and alleviating deficits in myelin regeneration in the mPFC (Pan et al. 2025).

In a rat model of vascular dementia, 4 weeks of treadmill exercise pretreatment significantly alleviated recognition memory impairment and anxiety‐like behavior. The physical exercise vascular dementia group demonstrated a higher density of dendritic spines and synapses intensity compared with the vascular dementia group in the mPFC. Moreover, treadmill exercise has been demonstrated to elevate dopamine and 5‐HT levels in the mPFC in comparison with vascular dementia controls (Zhang, Chen, et al. 2024). Furthermore, 3–4 weeks of aerobic exercise or strength training in aged Wistar rats enhanced spatial memory tasks, accompanied by an increase in synaptic plasticity proteins and neurotrophic signaling, including cAMP response element‐binding protein (CREB), BDNF, and p75 neurotrophin receptor. Additionally, aerobic exercise has been shown to increase the immunocontent of +NMDA receptor and PSD‐95 in the hippocampus, while reducing DNA damage. Conversely, strength exercise has been demonstrated to elevate the immunocontent of protein kinase C (PKC)‐α as well as the pro‐inflammatory factors TNF‐α and IL‐1β in the hippocampus (Vilela et al. 2017).

A further modulating effect of physical exercise on cognitive decline and hippocampal atrophy appears to be through the inhibition of GSK‐3. A 5‐month treadmill exercise regimen resulted in the inhibition of GSK‐3, which in turn led to a reduction in Aβ deposition and tau phosphorylation in the hippocampus in mice harboring expressing a chimeric mouse/human amyloid precursor protein and a mutant human presenilin 1 (APP/PS1) (Liu et al. 2013). The activation of the phosphatidylinositol 3‐kinases (PI3K)/Akt signaling pathway by treadmill running exercise from postnatal day 21 to postnatal day 34 has been demonstrated to result in the inhibition of GSK‐3β in socially isolated rats (Wang and Baek 2018). In addition, 8‐week treadmill running exercise was observed to reduce apoptosis and improve cognitive function, through an increased number of PI3K‐ and p‐Akt‐positive cells and an increase in the expression of PI3K, phospho‐Akt, and B‐cell lymphoma 2 (Bcl‐2) proteins. The expression of GSK‐3β and Bcl‐2–associated X protein (Bax) proteins was found to be decreased in the hippocampus of D‐galactose AD mice model (Y. Peng et al. 2022). The activation of the PI3K/Akt/GSK‐3 pathway by exercise is of significant importance, as lower levels of Akt activation result in a weakening of the inhibition of GSK‐3 (Hooper et al. 2008). Voluntary running wheel has been demonstrated to enhance the spatial memory of 1‐month‐old mice by modulating the cholinergic system, antioxidant activities, and apoptosis factors. This is achieved through an increase in antioxidant activity (increase catalase and decrease malondialdehyde), an increase in ACh, a reduction in apoptosis proteins, and the upregulation of the BDNF/PI3K/Akt/CREB pathway in the hippocampus (Wan et al. 2024).

##### 3.2.2.3 The Importance of Irisin in the Beneficial Effect of Physical Exercise on Cognition

Evidence indicates that physical exercise enhances neurogenesis by upregulating the proliferator‐activated receptor gamma coactivator‐1α (PGC‐1α)/FNDC5/irisin pathway. Irisin is a cleaved form of FNDC5 that is secreted into the bloodstream and can cross the blood–brain barrier and reach the brain (de Freitas et al. 2020; de Souza et al. 2023). It has been demonstrated that physical exercise increases *Fndc5* expression in the mouse hippocampus, resulting in *Bdnf* expression in a mechanism dependent on PGC‐1α (Wrann et al. 2013).

A recent study also indicates that irisin plays a pivotal role in regulating cognitive function in the context of physical exercise. This is evidenced by the observation that genetic deletion of *Fndc5/irisin* in mice results in impaired cognitive function in the context of exercise, aging, and AD. Additionally, the administration of peripheral irisin has been demonstrated to result in the enrichment of central irisin, which is sufficient to improve both the cognitive deficit and neuropathology in AD mouse models (Islam et al. 2021). In addition, studies have demonstrated that FNDC5/irisin levels are diminished in the hippocampus and cerebrospinal fluid of individuals with AD, and in elderly individuals with depression (Gonçalves et al. 2023; Lourenco et al. 2019).

Similarly, irisin levels were found to be diminished in the hippocampus of mice harboring the Swedish APP mutation and deletion of exon 9 of presenilin‐1 (APPswe/PS1dE9) and wild‐type mice that have been administered soluble Aβ, representing two distinct models of AD (Lourenco et al. 2019). In humans, decreased cerebrospinal fluid and serum irisin levels were observed in patients with AD and MCI compared with subjective memory complaints. A positive correlation has been found between cerebrospinal fluid and serum irisin levels, in global cognitive efficiency, as well as with specific cognitive domains, including memory, executive functions, attention, visuospatial abilities, and language (Pignataro et al. 2025). It is noteworthy that the restoration of central or peripheral irisin levels, achieved either through physical exercise or molecular manipulation, resulted in the enhancement of synaptic plasticity and memory in mouse models of AD (Lourenco et al. 2019). In the same way, an increase in peripheral and central irisin levels through swimming exercise has been shown to improve chronic cerebral hypoperfusion‐induced memory impairment, motor function, and anxiety‐ and depression‐like behaviors. This improvement is achieved through the inhibition of hippocampal neuronal apoptosis and microglial activation (Xiao et al. 2025).

##### 3.2.2.4 Contradictory Evidence Regarding the Effect of Physical Exercise on Cognition

The available evidence from human and animal studies indicates that physical exercise has a positive impact on hippocampal volume and neurogenesis. However, the results of some studies are not aligned with those of others. A systematic review observed modest effects of physical exercise on specific regions of brain gray matter volume (Hvid et al. 2021). However, some findings in this systematic review indicated that physical exercise had no effect or a small effect size, and the inconclusive nature of the effects of physical exercise was also noted (Hvid et al. 2021). In a separate original study, a 16‐week aerobic exercise intervention had no impact on brain volume or cognitive function in patients with AD (Frederiksen et al. 2018). Similarly, another study has demonstrated that aerobic training does not significantly impact hippocampal volume in cognitively unimpaired, healthy older individuals (Balbim et al. 2023). The varying results regarding the effect of physical exercise on hippocampal volume and cognition may be attributed to the heterogeneity of the study samples and the presence or absence of certain conditions, such as dementia and/or depression, in addition to cognitive deficits.

#### 3.2.3 Physical Exercise and Neuroinflammation in Dementia and Depression

It has been proposed that physical exercise protects against chronic and acute inflammation. This effect is achieved through the ability to enhance the production and release of myokines into the circulation, which may account for its anti‐inflammatory effect. Physical exercise has been demonstrated to induce the production of anti‐inflammatory cytokines, such as the classic IL‐10, as well as IL‐6 released by muscle fibers via a TNF‐α independent pathway. In addition, low to moderate physical exercise has been shown to inhibit the production of pro‐inflammatory mediators, such as TNF‐α, IL‐1β, IL‐18, CRP, and NLRP3 inflammasome (Pedersen 2019; Petersen and Pedersen 2005; Ross et al. 2019).

The impact of physical exercise on inflammatory processes is subject to the specific characteristics of the exercise regimen, including its intensity and duration, as well as the individual characteristics of the subject. While moderate‐intensity exercise has been demonstrated to have anti‐inflammatory effects, high‐intensity exercise has been shown to induce inflammatory responses (Wang et al. 2023). In healthy humans, acute exercise has been observed to induce a transient elevation in pro‐inflammatory markers. However, long‐term exercise may potentially lead to a reduction in pro‐inflammatory markers and an increase in anti‐inflammatory markers through the action of modulatory adaptations (Cerqueira et al. 2020). A single bout of maximal aerobic exercise has been observed to elicit a comparable increase in inflammatory cells (leukocytes, granulocytes, lymphocytes, and monocytes) in subjects with depression and healthy controls (Boettger et al. 2010). Similarly, another study that performed a maximal‐workload exercise challenge demonstrated a significant increase in plasma levels of IL‐8, IL‐6, and TNF‐α, with a concomitant decrease in IL‐4, in both depressed and control groups (Hallberg et al. 2010).

Conversely, it has been proposed that moderate‐intensity exercise represents the optimal intensity for the promotion of mental health, given that it has been observed that moderate continuous training results in a reduction in depressive symptoms and TNF‐α serum levels in university students. While HIIT has been demonstrated to reduce depressive symptoms, it has also been observed to increase perceived stress, TNF‐α, and IL‐6 relative to moderate continuous training (Paolucci et al. 2018). Other evidence in older adults indicated that high levels of physical exercise (> 180 min/week) were associated with lower CRP, IL‐6, and TNF‐α levels. On the contrary, among non‐exercisers, higher levels of other physical activity (e.g., household, walking and stair climbing, recreation, and occupational, volunteer, or caregiving activities) were related to lower levels of CRP and IL‐6 (Colbert et al. 2004). In elderly patients with MCI, a combination of aerobic exercises followed by strengthening, flexibility, and balance exercises, with or without cognitive training reduces levels of IL‐1β and IL‐6, whereas physical exercise combined with cognitive training reduces TNF‐α, Αβ40 and total tau levels (Katsipis et al. 2024). A systematic review evaluating the impact of physical exercise modalities to reduce pro‐inflammatory cytokines showed that moderate‐ to high‐intensity multimodal training (three times/week) in elderly with MCI and both voluntary cycling training and moderate or high‐intensity aerobic exercise in elderly with mild AD have an anti‐inflammatory effect. In addition, the same study also showed that low‐intensity treadmill training or high‐intensity swimming training inhibits pro‐inflammatory cytokines secretion in the rodent AD phenotype model (Ayari et al. 2023).

In vivo studies in rodents also corroborate the notion that physical exercise plays a pivotal role in regulating inflammatory processes. A study that transferred plasma from male mice subjected to voluntarily running for 28 days to non‐exercising mice showed that this strategy recapitulated the beneficial effects of physical exercise on neurogenesis and increased the contextual learning and memory of mice in the fear conditioning paradigm and improved their performance in the Morris water maze. Moreover, these infusions reduced LPS‐induced neuroinflammation in the hippocampus. The depletion of clusterin, a complement inhibitor protein, abrogated the anti‐inflammatory properties of plasma from exercised mice (De Miguel et al. 2021). Similarly, another study evaluated the process of microglial activation through the complement pathways in a mutant mice model of neurodegenerative diseases that overexpress mutant human TAR DNA binding protein 43 kDa in the primary motor cortex. An early intervention of 2 weeks of treadmill exercise training at the presymptomatic stage successfully inhibited microglial activation and parvalbumin interneurons engulfment. These effects were accompanied by the suppression of C6b‐9 levels and an upregulation of clusterin. The suppression of C6b‐9, as well as upregulation in clusterin, plays a role in inhibiting the complement pathway (Wei et al. 2023). Interestingly, dementia has been associated with the activation of complement pathways.

Resistance training also modulates inflammatory changes in the 3xTG mice model of AD through the reduction of TNF‐α mRNA levels in the frontal cortex and an increase in IL‐10 mRNA levels in the hippocampus. In addition, resistance exercise also decreases the number of positive ionized calcium‐binding adaptor molecule 1 (Iba‐1) microglia cells in the frontal cortex and the DG region of the hippocampus (Liu et al. 2020). Treadmill running exercise also reduces the mRNA levels of Iba‐1 and CD68 in the mPFC, both associated with microglial activation. These changes are accompanied by a reduction in the mRNA level of TNF‐α and an increased mRNA level of IL‐10 in the mPFC in the CUS‐induced depressive‐like behavior mice model (Liu, Xiao, et al. 2025). Other evidence also demonstrates the capability of physical exercise to facilitate a shift in the microglia from a pro‐inflammatory to an anti‐inflammatory state. This shift leads to a reduction in the production of pro‐inflammatory factors (TNF‐α and IL‐1β) and an increase in the secretion of anti‐inflammatory factors (IL‐10 and transforming growth factor beta), thereby promoting Aβ clearance and neural repair (Han et al. 2023; Hu et al. 2024; Liu et al. 2024; Sasaki et al. 2024). Treadmill running exercise has been demonstrated to ameliorate CUS‐induced depressive‐like behavior and effectively reverse the alterations in microglia in the mPFC in a rat model. Furthermore, treadmill running reduced the mRNA levels of Iba‐1, CD68, and TNF‐α, and increased IL‐10 in the mPFC in mice (Liu, Xiao, et al. 2025).

In an AD model induced by the intracerebroventricular administration of Aβ1–40, 4 weeks of treadmill running exercise prevented the development of depressive‐like behavior and the Aβ1–40‐induced increase in Iba‐1, thioredoxin interacting protein (TXNIP), and activation of the NLRP3 inflammasome in the hippocampus (Rosa et al. 2021). In fact, physical exercise appears to present a prophylactic effect. A study conducted by Zhang, Wang, et al. (2024) shows that 3 months of swimming exercise, starting from 1 month old, curbs cytokine response and mitigates sepsis through the decrease in serum TNF and IL‐1β levels, and reduction of infiltration of inflammatory cells in the liver, in male mice exposed to LPS challenge after an 11‐month interval of detraining. Prophylactic endurance exercise has been demonstrated to prevent an increase in P2X7R density, NLRP3, Caspase‐1, IL‐6, IL‐1β, and TNF‐α in the hippocampus and PFC of male Wistar rats (Miron et al. 2023). In a recent study conducted by Zhang, Li, et al. (2025), irisin was used against LPS‐induced inflammatory cognitive impairment in mice. The administration of irisin enhanced cognitive function and learning, accompanied by a reduction in microglial activation and a decrease in the expression of the NLRP3 inflammasome signaling pathway in LPS‐treated mice. In vitro data demonstrated that irisin suppressed microglial activation and reduced the production of pro‐inflammatory cytokines in BV2 cells exposed to LPS. Furthermore, irisin has been shown to confer protection against LPS‐stimulated BV2 microglia‐induced neurotoxicity in PC12 cells, while concurrently exerting an anti‐apoptotic effect in PC12 cells exposed to BV2 conditioned medium (Zhang, Li, et al. 2025).

In addition, the relationship between GSK‐3 and neuroinflammation is well described. GSK‐3 acts by upregulating the production of pro‐inflammatory cytokines (such as IL‐1β and IL‐6) and downregulating the production of anti‐inflammatory cytokines (Hoffmeister et al. 2020). In this context, the capacity of physical exercise to inhibit GSK‐3 activity may play an important role in the management of inflammation in dementia and depression (Peng et al. 2022). As previously discussed, physical exercise appears to modulate the inflammatory homeostasis within the CNS, thereby potentially preventing or treating the increase in pro‐inflammatory compounds in both the hippocampus and PFC. This suggests that physical exercise may serve as a strategy for modulating inflammation in dementia and depression.

## 4 Conclusion and Future Directions

The relationship between dementia and depression has been the subject of considerable research, with a growing body of evidence pointing to the existence of shared pathways between the two disorders. Furthermore, studies have identified depression as a risk factor for the onset of dementia. While there is currently no cure for dementia, emerging research has indicated that its onset can be mitigated by modulating various mechanisms that are still being elucidated. Late‐life depression has been identified as a contributing factor to 4% of dementia cases, while physical inactivity has been linked to 2% of cases. It has been demonstrated that regular physical exercise has the potential to not only prevent dementia and depression, but also to modulate the modifiable risk factors that may contribute to a delay in the onset of dementia. These risk factors include high LDL cholesterol levels, obesity, diabetes, and hypertension, which contribute to 7%, 1%, 2%, and 2% of dementia onset, respectively (Farina et al. 2024; Livingston et al. 2020; Pedersen and Saltin 2015).

**FIGURE 2:**
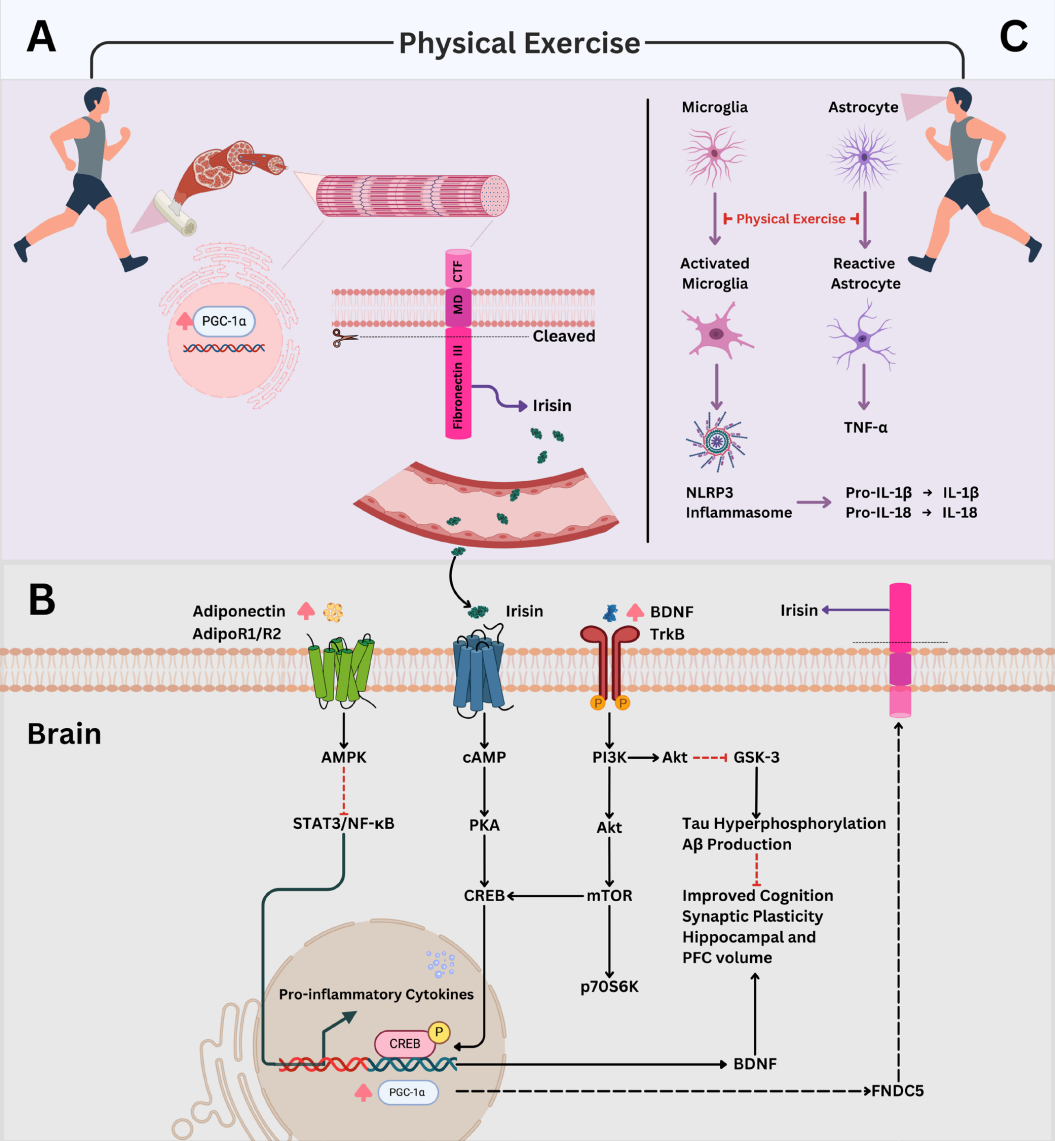
Physical exercise‐induced metabolites and signaling pathways in the brain. In skeletal muscle, physical exercise induces the expression of PGC‐1α which leads to increased production of FNDC5, which in turn is cleaved into irisin that is released into the bloodstream (A). Irisin can cross the blood–brain barrier and activate its receptor, inducing the downstream cAMP/PKA/CREB pathway, which leads to the production and elevation of BDNF levels in the central nervous system (CNS). BDNF interacts with TrkB receptor, activating the PI3K/Akt/CREB pathway, which induces BDNF production. In addition, the stimulation of the PI3K/Akt/mTOR/p70S6K pathway leads to the synthesis of synaptic proteins. The inhibition of GSK‐3 activity through phosphorylation (Ser9) mediated by PI3K/Akt pathway leads to the inhibition of both Tau hyperphosphorylation and β‐amyloid peptide production. In the CNS, an increase in adiponectin and activation of adipoR1/R2 leads to activation of the AMPK, resulting in the inhibition of NF‐kB/STAT3 pathway that decreases pro‐inflammatory cytokine production. Physical exercise also induces PGC‐1α expression, which in turn leads to an increase in FNDC5 and irisin in the CNS (B). In addition, physical exercise also can inhibit microglial activation and astrocyte reactivity in the CNS, thereby reducing neuroinflammation (C). Collectively, these physical exercise‐induced mechanisms contribute to synaptic plasticity, which in turn leads to improved cognitive function and increased hippocampal and PFC volume.

In this context, the evidence reviewed in this article leads to several general conclusions regarding the possible mechanisms by which physical exercise exerts a beneficial effect against dementia and depression, through modulation of shared mechanisms (Figure 2). Of particular significance are the observations that physical exercise has the capacity to modulate the HPA axis by regulating GCs release. Additionally, there is evidence that physical exercise can modulate neuroinflammation by decreasing pro‐inflammatory and increasing anti‐inflammatory cytokines. The modulation of the HPA axis and neuroinflammation leads to a reduction in Aβ and tau hyperphosphorylation, two features directly associated with the development of dementia. These physical exercise‐induced changes, together with the release of myokines (such as irisin), adipokines (such as adiponectin), and increased production of neurotrophins (such as BDNF), lead to an increase in hippocampal and PFC volume, promoting cognitive improvement. Given the beneficial effects of physical exercise on both dementia and depression, strategies should be developed to ensure that this practice becomes widespread, including through pharmacological strategies that mimic the beneficial effects of exercise to the population unable to practice it.

## Author Contributions

**Pedro Borges de Souza:** conceptualization, methodology, writing – original draft, writing – review and editing. **Ana Lúcia S. Rodrigues:** conceptualization, resources, supervision, writing – review and editing. **Fernanda G. De Felice:** conceptualization, resources, writing – review and editing.

## Conflicts of Interest

The authors declare no conflicts of interest.

## Peer Review

The peer review history for this article is available at https://www.webofscience.com/api/gateway/wos/peer‐review/10.1111/jnc.70185.

## Data Availability

Data sharing is not applicable to this article because no new data were created or analyzed in this study.
